# Viola Ovata for the Bite of the Rattle Snake

**Published:** 1834

**Authors:** 


8. *The Viola Ovata a remedy for the bite of the Rattlesnake.* Dr. S. W. Williams, of Deerfield, Massachusetts, on the authority of Dr. Wells of that state, has recommended this plant for the purpose above announced. It is the *Viola primulifolia* of Pursh; and described by Nuttall in the following terms:

"Leaves ovate, subcordate, crenate, rather acute, often lacerately toothed at the base; equally and for the most part conspicuously pubescent on either side, petiole marginated; scape shorter than the leaves; segments of the calyx subciliate; petals obovate, the two lateral ones bearded. On dry land. Flowers bright blue; flowers in April and May."

The following extract will present our readers with the results of Dr. Wells' observation and experience.

"Many years ago rattlesnakes abounded in the vicinity of this place. Since the land is cleared, they are rarely to be seen. Our old people were in the habit of using this violet for their bites. They generally know it by the name I have designated. The venerable Henry Wells, M. D., late of Montague, successfully employed it in these cases. To his statement respecting it I wish to draw the attention of physicians. He was called to a patient who was bitten by a rattlesnake, and who was laboring under all the symptoms of a diffusion of the venom. His body was enormously swollen, respiration laborious, and his skin livid. He immediately directed a strong infusion of the rattlesnake violet, and constantly bathed the wound and body with it. In a few hours the tumefaction subsided, the febrile symptoms abated, and the patient was considered nearly out of danger. He retired to rest, and left directions with the nurse to give the violet tea often during the night. The patient continued so much better that the nurse became negligent, and omitted the directions, and fell asleep. From this suspension of the remedy the patient relapsed, and the febrile symptoms returned, and the body was swollen like a puff-ball. The doctor was called, and again directed the remedy as before mentioned: the symptoms yielded, and from a continuance of the remedy two or three days he completely recovered without the use of any other means."

